# Pneumocystis Pneumonia in Patients with Autoimmune Diseases: A Retrospective Study Focused on Clinical Characteristics and Prognostic Factors Related to Death

**DOI:** 10.1371/journal.pone.0139144

**Published:** 2015-09-30

**Authors:** Minjiang Chen, Xinlun Tian, Fang Qin, Jiong Zhou, Jinjing Liu, Mengzhao Wang, Kai-Feng Xu

**Affiliations:** 1 Department of Respiratory Medicine, Peking Union Medical College Hospital, Beijing, China; 2 Department of Respiratory Medicine, China Meitan General Hospital, Beijing, China; 3 Offices for Infection Control, Peking Union Medical College Hospital, Beijing, China; 4 Department of Rheumatology, Peking Union Medical College Hospital, Beijing, China; Kliniken der Stadt Köln gGmbH, GERMANY

## Abstract

**Background:**

With the increasing use of immunosuppressive agents, the number of opportunistic infections has risen in patients with autoimmune diseases. Pneumocystis pneumonia (PCP) is one of these opportunistic infections that have a high mortality rate. However, only a few studies have described PCP in these patients, and these studies are limited in scope. We conducted this retrospective study to describe the clinical characteristics and factors associated with outcomes of PCP in patients with autoimmune diseases.

**Methods:**

A retrospective study was performed in laboratory diagnosed PCP patients with autoimmune diseases in an academic hospital over a 10-year period. Patients with human immunodeficiency virus (HIV) infection were not included. Clinical characteristics were collected and the factors related to death were analysed.

**Results:**

A total of 69 patients with PCP during the study period were included. Common clinical features included fever (81%), cough (56%), and dyspnea (35%). Ground glass opacity (81%) and reticulation (52%) were the most common radiological findings. Concurrent pulmonary infections including bacterium, aspergillus and cytomegalovirus were found in 34% of the patients. The overall in-hospital mortality rate was 32%. High mortality was associated with lower PaO_2_/FiO_2_ ratios and albumin levels. The lymphocyte count, CD4+ T cell count, previous usage of immunosuppressive agents, the duration and dose of glucocorticoids did not affect the outcome.

**Conclusions:**

The mortality rate in PCP patients with autoimmune diseases is high. Low PaO_2_/FiO_2_ ratios and albumin levels are independent prognostic factors of mortality.

## Introduction

*Pneumocystis jirovecii* (*P*. *jirovecii*) is an opportunistic fungal pathogen that causes pneumonia in patients with immunosuppressive diseases. At present, pneumocystis pneumonia (PCP) remains a leading cause of death in patients with human immunodeficiency virus (HIV) infection. However, due to the increased use of chemotherapy and immunosuppressive agents, the incidence of PCP in HIV-negative patients has also increased. Unlike PCP in HIV patients, non-HIV PCP has a different clinical course and shows a higher mortality rate [1,2,3,4]. Recently, there are many reports about PCP in patients with autoimmune diseases such as systemic lupus erythematosus (SLE) and rheumatoid arthritis (RA), especially in patients receiving prolonged usage of prednisone, immunosuppressive agents or biologic agents [5,6,7,8,9].

Several factors, such as female gender, co-infections, high D(A-a)O_2_, high lactate dehydrogenase levels, increased BUN, pre-existing lung disease, more severe pneumonia and delay of initial treatment, have been reported to be associated with poor prognosis in non-HIV patients with PCP [10,11,12]. However, the factors related to death in non-HIV PCP patients with autoimmune diseases still need to be defined. Thus, we conducted this study to describe the clinical manifestations and outcomes and to evaluate the prognostic factors related with death in Chinese patients from an academic hospital. Our study is the largest single-centre study focusing on non-HIV PCP patients with autoimmune diseases in Mainland China. The results will provide additional information on these characteristics in Chinese patients.

## Material and Methods

### Patients

Medical records of Peking Union Medical College Hospital (PUMCH, an academic hospital in China) between January 2004 and December 2013 were retrospectively reviewed. Eligible patients had laboratory confirmed PCP and were diagnosed with autoimmune diseases. Other eligible criteria included an age of at least 12 years and a negative serum HIV test. Written informed consent was obtained from all patients (or from a caregiver in the case of children) for their clinical records to be used in this study. This study was approved by the PUMCH ethics review board.

### Diagnosis

All cases of autoimmune diseases met the diagnostic criteria for their respective disease. The diagnosis of PCP was made on the basis of clinical symptoms and was confirmed by microbiological tests. Symptoms included one or more of the following: non-productive cough, dyspnea during exertion, fever, or chest pain. Computed tomography (CT) showed diffuse ground glass opacity (GGO), as well as inefficiency of antibiotics for non-PCP infections as previously described [13]. Respiratory specimens were obtained from bronchoalveolar lavage (BAL) fluid or hypertonic saline induced sputum. Tissue specimens were obtained from transbronchil lung biopsy or CT guided percutaneous lung puncture. A positive microbiological test was defined as the microscopic demonstration of *Pneumocystis jirovecii* through Gomorimethenamine silver (GMS) staining for the visualization of trophozoites and cysts in respiratory samples or lung tissue. Pneumocystis-specific nested polymerase chain reaction (PCR) was also performed in BAL specimens. PCP was confirmed if *Pneumocystis jirovecii* was found upon microscopic examination while positive results of PCR was regarded as infection only in patients with coinciding clinical symptoms of PCP, no evidence of other co-infections, and a response to anti-PCP therapy.

### Data collection

Demographical details, underlying autoimmune diseases, associated medical conditions, clinical characteristics, radiology manifestations and laboratory results were retrospectively reviewed. Use of immunosuppressive agents, glucocorticoids and biological agents before the diagnosis of PCP were summarized. The corticosteroid dose was expressed as the prednisolone equivalent dose. Laboratory values included the cytomegalovirus (CMV) PCR results, and findings from bacterial, fugal and tuberculosis cultures from respiratory samples were also analysed.

### Statistics

Descriptive statistics were used to calculate the means, standard deviations and frequencies. A univariate binary logistic regression analysis was performed. The continuous variables were analysed using the independent *t* test, and the categorical variables were compared by the *Chi*-square or Fisher’s test. *P*-values ≤0.05 were considered statistically significant. All covariates with a *P* value ≤0.05 were included in multivariate analysis.

## Results

Over the ten-year study period, a total of 264 patients were diagnosed with PCP in PUMCH. Among them, 69 patients with underlying autoimmune disease were included in this analysis.

### Demographic data and underlying conditions

The patients consisted of 25 (36%) males and 44 (64%) females with a median age of 39 years (range 13 to 78 years) at initial diagnosis. The most common underlying diseases were SLE (39/57%), followed by dermatomyositis/myositis (11/16%), vasculitis (9/13%), RA (6/9%), and other autoimmune diseases (4/6%). Comorbidities included kidney disease, interstitial lung disease, heart failure and diabetes mellitus. Among those with chronic kidney disease, two patients had undergone renal replacement therapy (Table 1).

**Table 1:** Demographical details, underlying diseases, and diagnostic procedures of the patients.

Variables	No. of patients (%) or median (range)
Age, median years (range)	39(13–78)
Gender	
Male	25 (36%)
Female	44(64%)
Underlying disease	
Systemic Lupus Erythematosus (SLE)	39(57%)
Dermatomyositis/Myositis	11(16%)
Vasculitis*	9(13%)
RA	6(9%)
Other CTDs^**^	4(6%)
Use of glucocorticoid	69(100%)
Prednisone	39(57%)
Methylprednisolone	28(41%)
Other glucocorticoids	2(3%)
Dose of glucocorticoid	49(5–133)
Duration of glucocorticoid	120(10–2670)
Use of immunosuppressive agents^#^	63(91%)
Cyclophosphamide	40(63%)
Cyclosporin A	8(13%)
Mycophenlatemofetil	7(11%)
Methotrexate	6(10%)
Tripterygium Glycosides	7(11%)
Acetazolamide	2(3%)
Use of biological agents^&^	5 (4%)
≥ 2 immunosuppressive agents	11(17%)
Diagnostic procedure	
BAL	31 (45%)
Sputum	40 (58%)
Tissue	2 (3%)
Positive GMS stain	38 (55%)
Positive PCR	45 (65%)
Co-infections	24(34%)

* Vasculitis: Behcet’s disease, microscopic polyangiitis, granulomatosis with polyangiitis

** Other CTDs: Sjogren syndrome (SS), undifferentiated connective tissue disease (UCTD), mixed connective tissue disease, scleroderma

# Immunosuppressive agents: cyclophosphamide, cyclosporin A, mycophenlatemofetil, and tripterygium glycosides

& Biological agents: ritaximab, and antitumor necrosis factor α(infliximab, entanercept)

### Immunosuppressive agents

Glucocorticoid was used in all patients. The mean dose was equivalent to 49±25 mg (range 5 to 133mg) prednisone. The median duration of usage was 120 days (range 10 to 2670 days). In the majority of patients (63/91%), immunosuppressive agents were used 3 months before PCP was diagnosed. The most commonly used immunosuppressive agent was cyclophosphamide (40/63%). Biological agents were used in 5 (4%) patients (including rituximab in 2 patient and tumor necrosis factor alpha inhibitors in 3 patients).

### Clinical, Radiologic and laboratory findings

The common clinical features included fever (56/81%), cough (39/56%), and dyspnea (24/35%). CT scans were performed in all 69 patients. The most common CT findings at initial diagnosis were ground glass opacity (56/81%) and reticulation (36/52%). The mean white blood cell count was 7335±4528 cell/μl, while the mean total lymphocyte count was 529±661 cell/μl. T cell subsets detection was performed in 38 patients. The mean CD4 positive T cell counts were 169±196 cell/μl. Serum albumin (ALB), immunoglobulin, and lactate dehydrogenase levels were also tested (Table 2). Microorganisms found in respiratory specimens other than *P*. *jirovecii* included bacterium (5 patients) and *aspergillus* (7 patients). CMV had been detected in 13 patients, including 9 patients with positive qualitative detection of CMV DNA by PCR and10 patients with positive CMV IgM.

**Table 2:** Clinical manifestations, Radiologic characters and laboratory findings of the patients.

	No. of patients (%)
Clinical manifestations	
Fever	56 (81%)
Cough	39 (56%)
Dyspnea	24 (35%)
Radiologic characters (CT scanning)	
Ground glass opacity (GGO)	56 (81%)
Reticulation	36 (52%)
Lymph node enlargement	25 (36%)
Patchy scattered infiltrates and parenchymal consolidations	22 (32%)
Pleural infusion	17 (25%)
Interlobular septa thicken	16 (23%)
Nodular consolidations	10 (14%)
Pneumothorax	5 (7%)
Cystic lesions	2 (3%)
Laboratory findings	
WBC count, cell/μl, mean ±SD	7335±4528
Lymphocytes, cell/μl, mean ±SD	529±661
CD4+ T cell, cell/μl	169±196
Albumin, g/L, mean ±SD	30±4
Serum IgG, g/L, mean ±SD	9.44±5.24
Serum IgA, g/L, mean ±SD	1.90±0.75
Serum IgM, g/L, mean ±SD	1.02±0.43
LDH, U/L	477±145
PaO_2_/FiO_2_ ratios, mean ±SD	217±98
Co-infections	24(34%)
Cytomegalovirus DNA PCR positive	13(19%)
Bacterium in BAL*	5(7%)
*Aspergillus* in BAL	7(10%)

* Included *pseudomonas aeruginosa* in three specimens, *Acinetobacter baumannii* in one specimen, *Klebsiella pneumonia* in one specimen

### Treatment and outcomes

**Fig 1:**
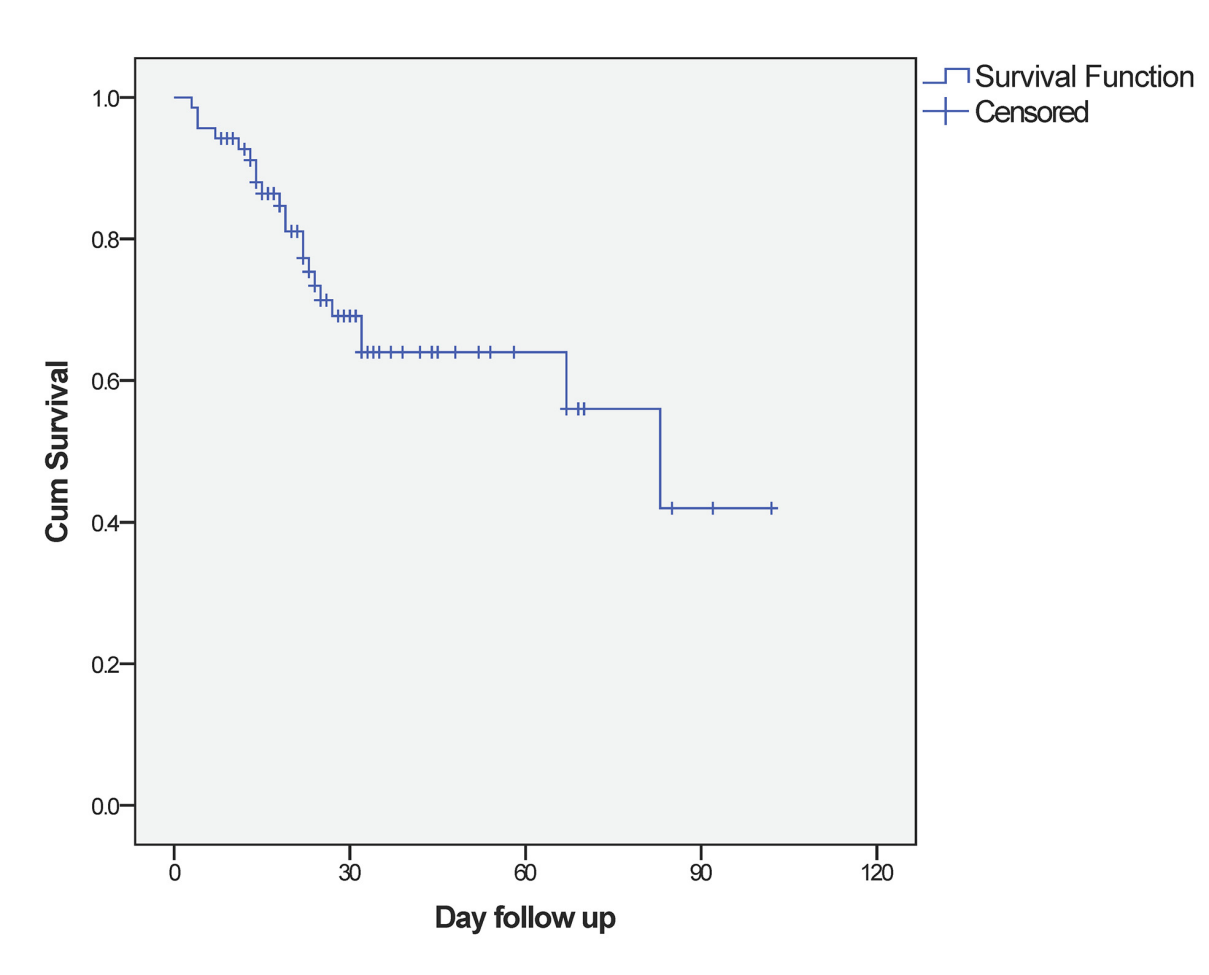
Kaplan-Meier survival curve for the patients with pneumocystis pneumonia.

Sulfamethoxazole (TMPco) was used as the first-line treatment in 59 (86%) patients. Among the 59 patients, 7 (10%) patients did not respond to TMPco. Clindamycin, primaquine and/or caspofungin were used as alternative agents. Patients with concurrent infections of CMV, bacteria or *aspergillus* were administered ganciclovir, cephalosporin, and itraconazole or voriconazole at the same time. Adjunctive corticosteroid was used in 58 (84%) of these patients. During the treatment period, a total of 23 (33%) patients developed shock. Of these patients, 20 (29%) required mechanical ventilation (MV), and 5 (7%) patients presented with pneumothorax. The 30-day in-hospital mortality rate was 26%, and the overall in hospital mortality rate was 32% (Fig 1). The deaths of 21 patients were attributed to PCP. One patient died from gastrointestinal bleeding.

### Prognostic factors related to death

The univariate comparisons of the factors between survivors and non-survivors were shown in Table 3. There were no significant differences between age, gender, comorbidities, previous usage of immunosuppressive regiments, duration and dose of glucocorticoids, immunoglobulin (Ig) levels, and lactate dehydrogenase (LDH) levels. The lymphocyte count (575±766 cell//μl vs. 423±304 cell//μl among survivals and non-survivals, respectively, *P* = 0.481) and CD4+ T cell count (182±209 cell//μl vs. 122±145 cell//μl, *P* = 0.236) were higher in the surviving patients, but the results were not statistically significant. The rates of initial TMPco treatment failure in the survival and non-survival group were also not significantly different. A low PaO_2_/FiO_2_ ratio at diagnosis (248±97 vs. 152±62, *P* = 0.000), shock during treatment (19% vs. 64%, *P* = 0.001), and use of MV (13% vs. 64%, *P* = 0.000) was related to poor prognosis. In addition, the low blood albumin level (32±4 g/L vs. 27±4 g/L, *P* = 0.030) and co-infection with *aspergillus* (4% vs. 27%, *P* = 0.011) were also associated with poor prognosis.

**Table 3:** Univariate analyses of risk factors among PCP patients determining survival rates.

Variables	Patients No. (%)		
	Survivors (n = 47)	Non-survivors (n = 22)	*P* value
Age, median years (range)	39(13–75)	41(14–78)	0.499
Male gender	18(38%)	7(32%)	0.498
Comorbidity			
Chronic renal disease	19(40%)	7(32%)	0.374
Idiopathic pulmonary fibrosis	3(6%)	4(18%)	0.140
Heart failure	2(4%)	0	1.000
Diabetes mellitus	5(11%)	3(14%)	0.703
Immunosuppressive agents	45(96%)	18(81%)	0.077
Previous days	319±751	196±422	0.731
Biological agents	4(9%)	1(5%)	1.000
Glucocorticoids dose (mg/d), mean ±SD*	49±22	49±34	0.779
Previous days	391±815	312±453	0.960
Severity of illness			
PaO_2_/FiO_2_ ratios, mean ±SD	248±97	152±62	0.000
Shock	9(19%)	14(64%)	0.001
Mechanical ventilation	6(13%)	14(64%)	0.000
Pneumothorax	1(2%)	4(18%)	0.033
Laboratory findings			
WBC count, cell/ul, mean ±SD	6753±4043	8670±5380	0.338
Lymphocytes, cell/ul, mean ±SD	575±766	423±304	0.481
CD4+ T cell, cell/ul	182±209	122±145	0.236
Albumin, g/L, mean ±SD	32±4	27±4	0.003
Serum IgG, g/L, mean ±SD	9.66±5.66	8.82±4.14	0.909
Serum IgA, g/L, mean ±SD	1.76±0.66	2.36±0.90	0.153
Serum IgM, g/L, mean ±SD	1.07±0.45	0.88±0.37	0.354
LDH, U/L	488±148	563±213	0.264
Co-infections			
CMV	9(19%)	4(18%)	1.000
Bacterium	3(6%)	2(9%)	0.651
*Aspergillus*	2(4%)	6(27%)	0.011
Treatment			
Adjunctive steroid therapy	39(85%)	19(86%)	1.000
Use of TMPco	42(89%)	17(77%)	0.271
Not respond to TMPco	3(6%)	4(18%)	0.191

* Corticosteroids doses were expressed as the prednisolone equivalent dose

In multivariate analysis, all covariates with a *P* value <0.05 (PaO_2_/FiO_2_ ratios, incidence of shock, MV, ALB level, *aspergillus* co-infection) were included in the regression analysis. The result showed that the PaO_2_/FiO_2_ ratios, mechanical ventilation, and ALB level were dependent predictors of mortality (Table 4).

**Table 4:** Multivariate analysis for Prognostic factors.

Variables	Adjusted OR	95%CI	*P* value
PaO_2_/FiO_2_ ratios	1.005	1.002–1.009	0.006
Incidence of shock	0.589	0.240–1.444	0.247
Mechanical ventilation	1.676	0.642–4.380	0.292
Albumin	1.089	1.002–1.183	0.044
*Aspergillus* co-infection	0.679	0.342–5.199	0.679

### Subgroup analysis of patients with SLE

Thirty-nine PCP patients with underlying SLE were analysed as a subgroup. The mortality rate in this group was 25.6% (10/39). Similar to the other CTD patients, a lower PaO_2_/FiO_2_ ratios (234±127 vs. 150±73, *P* = 0.014), incidence of shock (14% vs. 60%, *P* = 0.009), and usage of mechanical ventilation (7% vs. 80%, *P* = 0.000) were associated with poor prognosis in these patients. Interestingly, higher CD4+ T cell counts were found in the survival group (175±128 vs. 75±33, *P* = 0.024). Higher proportions of non-survivors were female, but no statistical significance was found (51% vs. 90%, *P* = 0.057).

## Discussion

PCP is an uncommon but fatal disease in patients with autoimmune disease. The clinical manifestations in these patients differ from those with HIV infection and the mortality rate is significantly higher (between 32% to 44% in previous reports) [8,14,15]. We accumulated the largest possible number of patients with CTD in China who developed PCP and analysed the symptoms, clinical courses, and prognosis factors in these patients. To our knowledge, this is the largest study performed on HIV-negative PCP patients with underlying autoimmune diseases.

In our study, all patients were under glucocorticoid therapy and most of them combined this with immunosuppressive agents. The most common patient radiologic features identified through CT scanning were ground glass opacity and reticulation [16]. The laboratory result showed that the lymphocyte count, CD4+ T cell count, and serum ALB were lower than normal and that the LDH level was elevated. WBC count and Ig levels were normal in these patients. These findings were similar with the previous report [12]. The co-infection rate in our study was higher than that in previous studies performed in HIV negative patients [10]. We thought the disturbance of the immune system in these patients and the usage of immunosuppressive agents might have caused these patients to become more susceptible to infections.

The overall mortality rate in our study was 32%, which is similar to that found in previously published studies [14,8]. Previous studies have demonstrated that high D(A-a)O_2_ was associated with poor prognosis in non-HIV PCP patients [10,11]. In our study, PaO_2_/FiO_2_ ratios in the survival and non-survival groups were 248±97 vs. 152±62 respectively. The lower PaO_2_/FiO_2_ ratio, which was also a representative of ventilation-perfusion abnormality, was related to poor prognosis in CTD patients.

Hypoalbuminemia decreased plasma colloid osmotic pressure, which increased pulmonary vascular permeability and compromised the intravascular volume, placing the patient at risk for inadequate blood flow to vital organs. Previous studies showed that hypoalbuminemia had a positive correlation to increased lung injury and could be a significant indicator of mortality and morbidity in critically ill patients [17,18,19]. Lower ALB level is also reported to be associated with poor prognosis in PCP patients with CTD and HIV-negative patients [8,20]. But in a recent research performed by Kim et al., hypoalbuminemia was not considered as an independent predictor of mortality. In our study, the mean ALB level was higher in survivors than non-survivors. The difference was significant in both univariate and multivariate analysis. These findings suggest that ALB levels might be a predicting factor to be used in the diagnosis of PCP patients with CTD.

Incidence of shock and MV were reported to correlate with worse prognosis in non-HIV PCP patients [11]. In our study, the incidence of shock and MV rate also differed significantly in the survival and non-survival groups. These factors reflect the seriousness of diseases including organ failure, poor general condition and the need of intensive care, which made the patients more vulnerable to in hospital infections and other complications. Although these events were not identified as independent risk factors in multivariate analysis, more attention should be paid to patients with these conditions.

*Aspergillus* infection is one of the most common invasive fungal infections in SLE [21]. Patients with active disease and under high doses of corticosteroids (>60mg/day) are more vulnerable to *aspergillus* infection [21]. Because of the state of immunosuppression, mixed infection was common in these patients and the mortality rate was high. In our study, we also found that *aspergillus* co-infection was associated with higher mortality in univariate analysis. Due to the low incidence of combined *aspergillus* infection, the relationship was found to be not significant in multivariate analysis.

Despite presenting intolerance and adverse events, TMPco is still the first-line therapeutic regiment for *P*. *jirovecii* pneumonia [22,23,24]. The failure of initial TMPco treatment has been reported as a poor prognosis factor in HIV-negative patients. In our study, 86% of patients were initially treated with TMPco, and first-line therapy failure was observed in 12% of the patients. However, this study did not show any differences in the TMPco failure rate between the survival and non-survival groups. We hypothesized that this finding may be due to the limited sample size, intensive monitoring of the patient’s symptoms and a prompt change to the second-line regimen (clindamycin, primaquine and/or caspofungin).

As a retrospective study, our study has several limitations. First, the incidence of SLE in our study is much higher than the incidence in other CTDs in China. SLE was thus the most common underlying autoimmune disease in our study. Second, some patients were diagnosed by PCR based assay. This diagnostic procedure is more sensitive than microscopic examination but cannot distinguish between infection and colonization. However, we used only bronchoalveolar lavage fluids specimens and performed the test in patients with clinical symptoms and radiologically compatible manifestations of PCP, which may have elevated the positive predictive value and diagnostic accuracy of this procedure [25,26]. Third, because it was a retrospective study, some factors that may have influenced the outcome of PCP were unavailable, such as LDH and C-reactive protein. The pneumonia severity index, the treatment delay and disease activity of underlying CTD could not be evaluated well. Furthermore, our study was carried out in a single institution. As the largest rheumatic autoimmune treatment centre in China, our hospital may have CTD patients with diseases that are more serious and with more comorbidities. The selection bias may have influenced the significance of our findings. A future prospective study of Chinese patients with a larger sample size is still required.

## Conclusion

In summary, this study found that the mortality rate is high in PCP patients with underlying autoimmune disease in China. A lower PaO_2_/FiO_2_ ratios and ALB level were dependent predictors of mortality.

## Supporting Information

S1 Table Clinical characters and diagnostic procedure. (XLSX)

S2 Table Radiologic characters in CT scanning. (XLSX)

S3 Table Comorbidities, treatment and outcome. (XLSX)

S4 Table Laboratory findings and co-infections. (XLSX)
